# A Chromatographic Test Reveals Homeopathic Preparations Affect Human Blood *In Vitro*

**DOI:** 10.1055/a-2748-6796

**Published:** 2026-05-13

**Authors:** Daniel Wrzałko, Jürg Lehmann, Stephan Baumgartner, Ursula Wolf, Maria Olga Kokornaczyk

**Affiliations:** 1 Institute of Complementary and Integrative Medicine, University of BernBernSwitzerland; 2 Hiscia Research Institute, Society for Cancer ResearchArlesheimSwitzerland; 3 Private Practice for General Internal MedicineBaselSwitzerland; 4 Institute for Integrative Medicine, University of Witten/HerdeckeHerdeckeGermany

**Keywords:** individualised homeopathy, human blood model, ascending paper chromatography, pattern evaluation, Kaelin blood test

## Abstract

**Background:**

Classical homeopathy is a medical system with individualised treatment of patients. An ascending paper chromatography test, known as the Kaelin blood test, was adapted to investigate the effects of homeopathic preparations on human blood *in vitro* with an emphasis on possible inter-individual differences in response rate.

**Methods:**

Thirteen patients to whom homeopathic remedies had been prescribed were enrolled in the study; EDTA blood samples were collected from each of them prior to remedy intake. Portions of each sample were treated *in vitro* with the patients' individually prescribed remedy, *Pulsatilla* , *Natrium muriaticum* at either 30c or 200c potency, or placebo. All samples were analysed using the Kaelin blood test. To assess the stability of the experimental set-up, 13 analogous systematic negative control (SNC) experiments were performed. Additionally, 16 untreated blood samples were analysed to correlate the chromatographic pattern features with blood parameters. Chromatograms from both experimental series were scanned and evaluated using computerised image analysis.

**Results:**

Two-way analysis of variance with *patient* and *treatment* as independent factors revealed statistically significant interactions ( *p* < 0.05) in 10 out of 10 pattern evaluation parameters; only three parameters showed significant main effects ( *p* < 0.05). The SNC experiments did not show any statistically significant effects, being indicative of a robust and stable experimental set-up. These findings suggest that the response of the blood samples to the homeopathic treatments was contingent upon the individual patient, indicating patient-specific effects. Strong correlations were found between pattern features and blood parameters related to haemoglobin and liver function.

**Conclusion:**

The Kaelin blood test may be considered a potentially useful *ex vivo* , *in vitro* model for investigating individualised responses of human blood samples to homeopathic treatments.

## Introduction

Homeopathy, a medical system developed by Samuel Hahnemann in the 18th century, 1 relies on the 'Law of Similars'. This principle states that a substance that induces certain symptoms in a healthy person can cure similar symptoms in a sick person when administered in diluted form (often beyond the Avogadro limit). Although homeopathy is used worldwide, its mode of action is a point of discussion.

Analytical methods based on the self-organisation of matter into ordered structures are increasingly used in various fields of medical research, including diagnosis, 2 pharmaceutics 3 4 and basic homeopathy research. 5 6 7 8 These methods exploit the characteristics of the emerging patterns, such as their degree of order or complexity, to characterise and differentiate the tested samples.

In basic homeopathy research, the most common self-organisation-based methods involve droplet evaporation 3 6 9 10 and copper-chloride biocrystallisation, 5 7 8 which rely on the pattern formation induced by drying of fluids with complex composition. It was shown for instance that homeopathically treated and untreated plants 5 and seeds 7 delivered significantly different patterns when analysed using these methods. In other studies, the self-organised patterns revealed differences related to the manufacturing technique applied to produce the homeopathic preparations: for example, varying number of succussion strokes applied during potentisation 3 or the type of succussion movement employed (e.g., turbulent vs. laminar). 10 11 12

Another group of methods, also based on self-organisation of matter, involves paper chromatography. These methods utilise capillary forces as separation mechanisms, causing the tested solutions to migrate into the filter papers, creating patterns. 13 14 15 16 17 In contrast to commonly used chromatography aimed at achieving separation of the individual compounds or fractions within a sample, 18 the here-reported tests focus on creating complex and ordered patterns in which shapes and colours are used to differentiate between the samples. 19 Paper chromatography has been used, though less commonly, for analyzing the quality of soils, 13 agricultural products and foods 19 20 21 and medicinal plants. 17 There were also attempts to analyse animal and human body fluids. 21 Finally, some applications in the field of homeopathy basic research were also reported. 21 22

The present study employs the Kaelin blood test—a method based on ascending paper chromatography, in which blood haemolysate serves as stationary phase, and water as mobile phase. The test was developed in the 19th century by Dr. Werner Kaelin (1888–1973), an anthroposophical physician and co-founder of the Society for Cancer Research (Arlesheim, Switzerland). 23 In its original form, the test was intended to detect cancer. 24 25 26 27 In our study, we adapted the experimental protocol and applied this method to analyse the *in vitro* effects of homeopathic preparations on human blood.

This study investigated whether *in vitro* homeopathic treatments (individually prescribed remedies or *Pulsatilla* or *Natrium muriaticum* at either 30c or 200c potency, vs. placebo) induce differences in the chromatographic patterns obtained from blood samples. We also explored whether any observed differences were patient-specific and analysed correlations between blood parameters (blood count and clinical chemistry results) and pattern evaluation parameters. *Pulsatilla* and *Natrium muriaticum* were chosen as the two pre-determined remedies because, based on consultations with homeopaths, they are considered to have distinctly different remedy pictures. This contrast was expected to increase the likelihood that any observable effects would be captured in the chromatographic patterns. Moreover, in a pilot study (unpublished data), these remedies demonstrated promising potential for use in this type of investigation.

## Materials and Methods

### General Study Design

Our study analysed homeopathically treated and untreated EDTA blood samples using the Kaelin blood test across two independent experimental series.

#### Ethical Approval and Informed Consent

For experimental series I, ethical approval was obtained from the Ethics Committee of Northwest and Central Switzerland (EKNZ; approval no. 2022-01088). In contrast, as confirmed by a clarification of responsibility, experimental series II did not fall within the scope of the Swiss Human Research Act (HRA, Art. 2) and the EKNZ was not responsible for assessing this project.

All experiments were performed in accordance with relevant guidelines and regulations. Informed consent was obtained from each participant whose blood sample was used in experimental series I. For experimental series II, anonymised leftover laboratory samples were used. The study is not a clinical trial (clinical trial number: not applicable).

#### Investigation of the Impact of Homeopathic Remedies on Blood *in Vitro* (Experimental Series I)

In the first experimental series, 13 EDTA blood samples collected from 13 different patients were used. Each blood sample was processed individually. No pooling of blood occurred at any stage. Each sample was used for the performance of one verum experiment testing the influence of *in vitro* homeopathic treatments and one systematic negative control (SNC) experiment to assess the experimental system stability. Experimental series I thus comprised 26 experiments (13 verum and 13 SNCs). Each of the verum experiments compared the effects of four *in vitro* treatments on the blood (individually prescribed remedy [see section Materials and Methods], *Pulsatilla* , *Natrium muriaticum* , at either 30c or 200c potency, and placebo), whereas SNC experiments were conducted using only placebo. The treatments were tested in triplicate with three chromatograms per replicate, resulting in nine chromatograms per treatment within a single experiment. The verum and SNC experiments thus yielded a total of 936 chromatograms (26 experiments × 4 treatments × 9 chromatograms). The chromatograms were evaluated using computerised image analysis, and the obtained data were statistically analysed.

Outcome measures of the impact of the *in vitro* treatment on blood were the pattern evaluation parameters describing the sizes and shapes of particles visible on scanned chromatograms converted to binary images.

#### Investigation of Correlations between Blood Parameters and Pattern Evaluation Parameters (Experimental Series II)

Experimental series II employed the Kaelin blood test to analyse 16 untreated EDTA blood samples (four samples per experiment). Each sample was analysed in triplicate, generating three chromatograms per replicate. This resulted in a total of 144 patterns (16 samples × 3 replicates × 3 chromatograms). The pattern evaluation parameters were subsequently analysed for correlations with blood parameters.

### Experimental Series I

#### Blood Sample Collection in Experimental Series I

##### Patient Recruitment

Patient recruitment was performed from September to November 2022. J.L. (a qualified homeopathic physician) recruited participants from his private practice in Basel, Switzerland. Patients were provided with a patient information sheet. Those interested in participating signed the informed consent form. J.L. completed a paper case report form for each participant, capturing data on sex, age, diagnosis/condition, prescribed homeopathic remedy and its potency, chronicity or acuteness of the diagnosed disorder, type of prescription (constitutional or other), recent use of homeopathic remedies for the same condition and prior history of homeopathic treatment. J.L. applied a clinical (non-classical) homeopathic approach, selecting remedies based on a brief, symptom-oriented consultation rather than a full classical homeopathic interview. In the context of this study, such preparations are referred to as 'individually prescribed remedies' and were used as one of the four examined treatments.

Importantly, each individually prescribed remedy was applied exclusively to the blood sample originating from the respective patient; no individually prescribed remedy was used across samples from different donors.

#### Inclusion Criteria

This study enrolled adult patients who visited J.L.'s medical practice and received a homeopathic treatment prescription during that visit. The patients were eligible only if they had never taken any homeopathic remedy before the visit or had been taking a different remedy than the one prescribed.

#### Exclusion Criteria

The following factors excluded patients from participation: prescription of *Natrium muriaticum* or *Pulsatilla* , poor blood clotting, intake of medications that interfere with blood clotting (anticoagulants or antiplatelets), a serious medical condition where blood donation would pose an additional risk, age under 18 years, insufficient understanding of the patient information language or inability to provide informed consent for any other reason.

#### Blood Collection for Experimental Series I

Thirteen patients were enrolled in the study. Blood collection was performed during the visit, prior to the intake of the prescribed remedy, to ensure that the blood was not influenced by the remedy *in vivo* , allowing assessment of its effects solely *in vitro* . Samples from each patient were collected in two EDTA vacuum tubes: one for the verum experiment and one for the SNC experiment. The tubes were coded by the recruiting physician (J.L.) at the time of blood collection to ensure anonymisation at source. The experimenters received only coded samples and could not match the blood samples to individual patients. Case report forms were likewise linked only to the anonymised codes. Immediately following collection, the blood samples were frozen. They were then transported to the laboratories of the Society for Cancer Research in a heat-insulating polystyrene box and stored at −21°C until analysis. Along with the samples, globules of the prescribed remedies and completed case report forms were provided to the experimenters.

#### Homeopathic Remedies (Experimental Series I only)

For the *in vitro* treatments of experimental series I commercially available homeopathic globules were used (size 3; Spagyros AG, CH-3076 Worb, Switzerland). The four treatments were as follows: individually prescribed remedy, *Pulsatilla, Natrium muriaticum* , and placebo for verum experiments, and only placebo for SNCs. The individually prescribed remedy and its potency were selected by J.L. during consultation using a clinical homeopathic approach. Each individually prescribed remedy was applied only to the blood sample obtained from the respective patient and was not used in experiments involving samples from other donors. The potency levels of the pre-determined remedies ( *Pulsatilla* and *Natrium muriaticum* ) were aligned with the potency of the individually prescribed remedy for each patient and were either 30c or 200c. Placebo globules were not impregnated but were not discernible from the verum globules. The individually prescribed remedies tested alongside the two pre-determined remedies and placebo in the 13 verum experiments are listed in Table 1 .

**Table 1:** Information concerning patients included in experimental series I of the study

Patient	Gender	Age	Diagnosis ICD 10	Prescribed remedy	Chronic or acute state	Type of prescription	Any previous intake of homeopathic medicines	Previous intake of homeopathic medicines for current condition
1	F	70	R14 Flatulence and related conditions; K59.0 Constipation	*Nux vomica* 200c	Chronic	Constitutional	Yes	No
R2	M	57	S02.2 Fracture of nasal bones; S06.0 Concussion; F07.2 Post-concussional syndrome	*Nux vomica* 200c	Acute	Constitutional	Yes	No
3	F	34	M25.5 Pain in joint	S *ymphytum officinale* 200c	Chronic	Other	Yes	No
4	F	54	S00.9 Superficial injury of head, part unspecified; S40.0 Contusion of shoulder and upper arm	*Arnica montana* 30c	Acute	Other	Yes	No
5	F	24	R51 Headache; M54.2 Cervicalgia	*Gelsemium sempervirens* 30c	Chronic	Other	Yes	Yes
R6	F	70	R43 Disturbances of smell and taste	*Sulphur* 200c	Chronic	Other	Yes	No
7	F	34	H65.0 Acute serous otitis media	*Hepar sulphur* 30c	Acute	Other	Yes	Yes
R8	F	59	L29.8 Other pruritus	*Sulphur* 30c	Acute	Other	Yes	No
R9	F	57	R05 Cough; R06.0 Dyspnea	*Ipecacuanha* 30c	Acute	Other	Yes	No
10	F	66	I49.4 Other and unspecified premature cardiac depolarisation	*Lycopodium clavatum* 30c	Chronic	Other	Yes	No
R11	F	45	J06 Acute upper respiratory infections of multiple and unspecified sites	*Phosphorus* 30c	Acute	Constitutional	Yes	No
R12	F	70	K57 Diverticular disease of intestine	*Lycopodium clavatum* 30c	Acute	Other	Yes	No
13	M	51	K52.9 Non-infective gastroenteritis and colitis, unspecified	*Sulphur* 200c	Chronic	Constitutional	Yes	No

Notes: Donors whose samples responded to the treatments *in vitro* are marked with the letter ‘R’. F, female; M, male.

#### Blinding and Randomisation (Experimental Series I only)

Prior to the experiment (verum and SNC), a person not involved in the experimentation transferred the globules for each *in vitro* treatment from the original package to two letter-coded vials (one for the stationary phase and one for the mobile phase; see section 'The Kaelin blood test'). The person conducting the experiments was blinded to both the treatments and the order of the verum and SNC experiments. The Kaelin test randomisation design was consistent within each experimental pair (verum and SNC).

#### *In Vitro* Treatment (Experimental Series I only)

Thawed blood (from one EDTA test tube) was diluted with purified water according to Pharm.Eur. 9.4 ('purified water in bulk', X-SEPTRON LINE 10 VAL, BWT AQUA AG, Aesch, Switzerland) to a concentration of 7% (v/v). The resulting blood haemolysate was divided into 12 portions of 4 mL each (three replicates per each of the four treatments). To each blood haemolysate portion, three globules of the tested treatment were added. After the globules dissolved, the solutions were slightly mixed by hand to ensure a uniform distribution of the treatment. Solutions used for the mobile phase were prepared by adding three globules of the appropriate treatment to each of 12 tubes containing 7 mL of purified water and waiting for them to dissolve. Each of the 12 portions of the treated blood haemolysate and water served for the preparation of three chromatograms.

#### The Kaelin Blood Test

The Kaelin blood test ( Fig. 1 ) is an ascending paper chromatography test that consists of two phases: the stationary phase ( Fig. 1A–D ), in which blood haemolysate raises on a chromatographic paper and a mobile phase ( Fig. 1E–J ), in which water raises on the paper with the dried stationary phase, elevating and distributing the blood pigments into a specific pattern ( Fig. 1K–M ; Supplementary file 1 [a video], available in online version only).

**Fig. 1:**
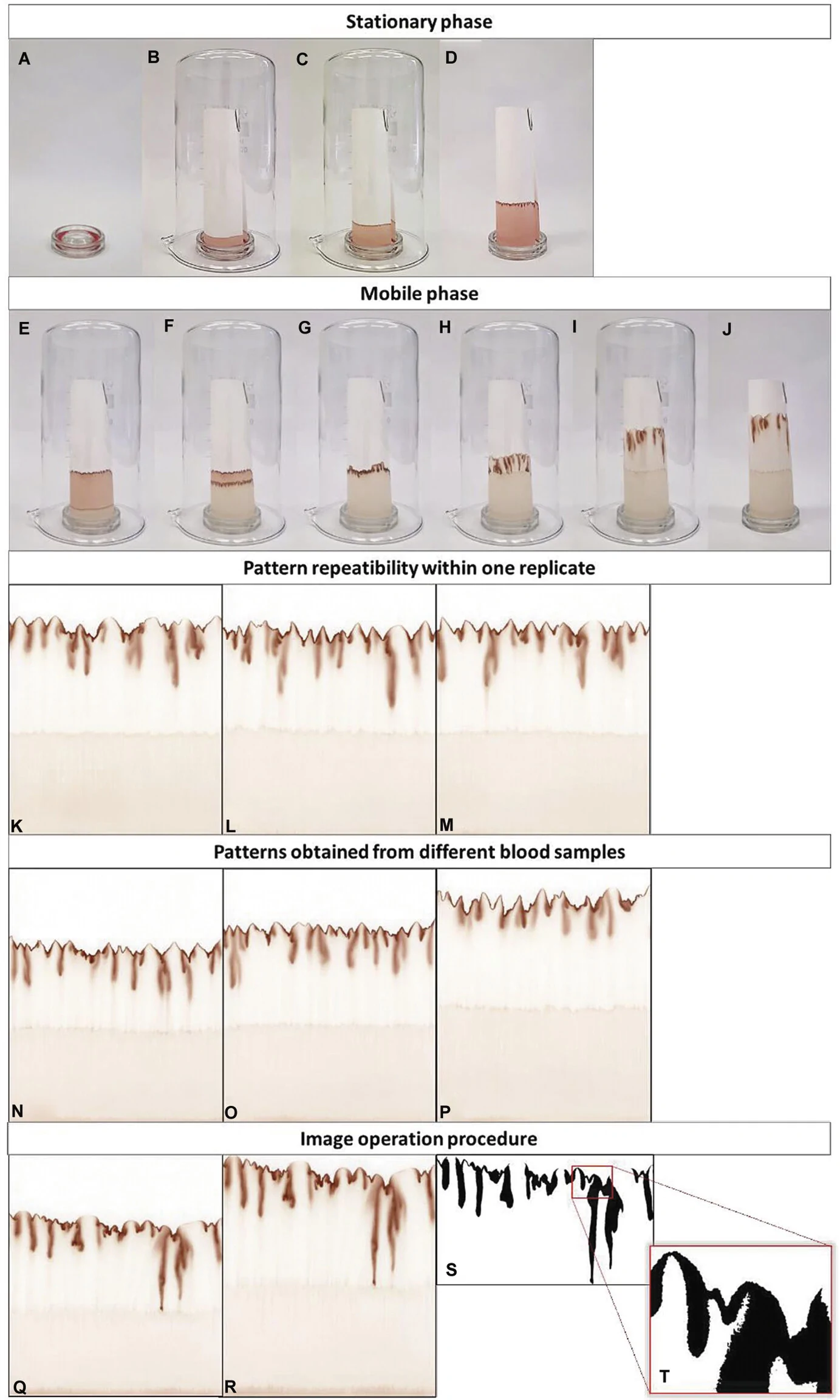
Graphical representation of experimental procedures conducted during the Kaelin blood test ( **A-J** ). Stationary phase: blood haemolysate ( **A** ) ascends along the chromatographic paper ( **B, C** ) and is allowed to dry ( **D** ). Mobile phase: water ascends along the residuum remaining after the stationary phase ( **E, F** ), breaks the borderline ( **G** ), rises further elevating the blood pigments and creating the target form ( **H, I** ); the final pattern is allowed to dry ( **J** ). Examples of three patterns obtained from one replicate showing repeatability ( **K–M** ) and from blood samples collected from three different patients presenting the variability of the heights and characteristics of the target forms ( **N–P** ). Steps of the image processing procedures: cutting the edges of the pattern ( **Q** ), scaling ( **R** ), conversion to binary and cropping to the applied region of interest ( **S** ); close-up of an image fraction showing the particles ( **T** ).

In the stationary phase, 1 mL of the *in vitro* treated blood haemolysate was pipetted into the glass Kaelin dish ( Fig. 1A ). A rolled sheet of chromatographic paper was placed upright on the dish containing the haemolysate. To maintain humidity, the dish was covered with a 1,500 mL beaker ( Fig. 1B ). The haemolysate was allowed to migrate up the paper for 20 minutes ( Fig. 1B, C ). Afterwards, the beaker was removed, and the paper was allowed to dry ( Fig. 1D ).

The mobile phase involved pipetting 2 mL of water treated with the same remedy as used in the stationary phase into the Kaelin dish. The same rolled sheet of chromatographic paper was then placed back on the dish. The beaker was used again to cover the dish and maintain a humid environment. The water was allowed to migrate up the paper containing the stationary phase residuum for 90 minutes ( Fig. 1E–I ). After this time, the beaker was removed, and the paper was allowed to dry ( Fig. 1J ).

#### Scanning of the Chromatograms

The chromatograms were scanned using the Epson Perfection V600 Photo scanner (Seiko Epson Corporation, Nagano-ken, Japan) with the *EPSON Scan* software. The scanning settings were: 24-bit colour type, 300 dpi resolution and 140.1 mm × 170.5 mm dimension. The resulting images (1654 × 2014 pixels) were saved in TIFF format for evaluation.

#### Image Evaluation

All image processing and analysis steps were performed using the software ImageJ (version 1.54f). 28 To minimise edge effects, a strip of pixels was cut off from each image and excluded from the analysis (66 pixels from the left, 184 pixels from the top, 88 pixels from the right and 46 pixels from the bottom); thus, the final image size was 1500 × 1784 pixels.

In a second step, the chromatographic patterns were aligned to the same height within each image. The topmost pixel of each pattern was identified, and the images were cropped at that level. The tallest pattern (1677 pixels) served as a reference, and all other images were scaled to match its height using bi-cubic interpolation to compensate for the added pixels.

After scaling, RGB images were split into their red, green and blue colour channel. RGB images as well as single-colour images were converted to binary.

A rectangular region of interest (ROI) was defined to encompass the dark red, waved form present in the upper region of the pattern (referred to as the target form; Fig. 2 ). To establish the ROI, the image with the lowest reaching target form was identified. This target form reached the 912th pixel row from the top. This row was set as the lower limit of the ROI containing the target form ( Fig. 1S ), which was then applied to all images. Within this ROI, particles of size 3 pixels to infinity were investigated. Particles smaller than 3 pixels were considered noise and excluded from the analysis.

**Fig. 2:**
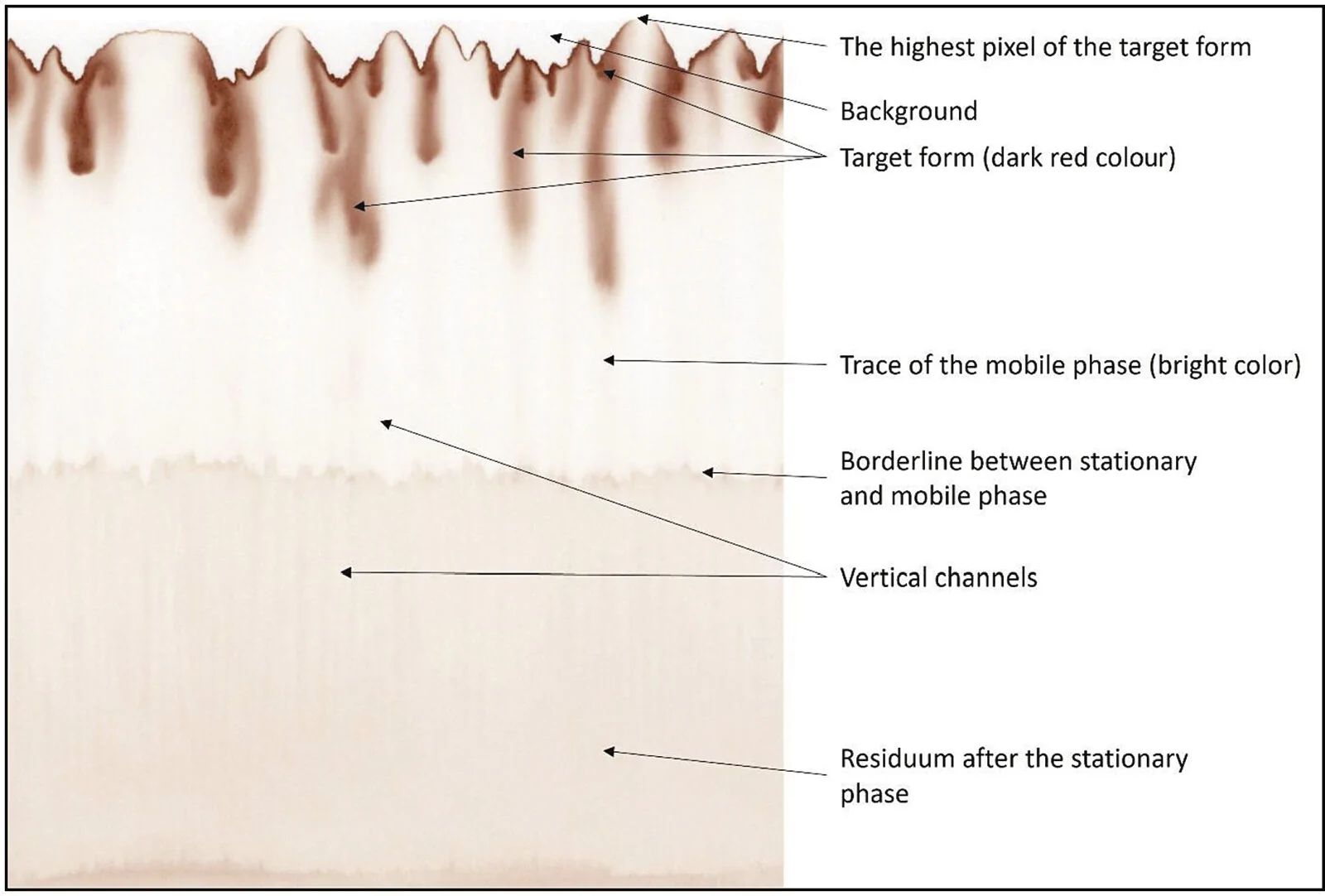
Example of a scaled chromatographic pattern with marked features that emerged during both phases of the Kaelin blood test.

As outcome measures, the following pattern evaluation parameters were assessed by means of the particle-analysing tool: number of particles (count), *average size* and *perimeter* of particles, *major* and *minor* axes, *circularity* , *solidity* , *Feret's diameter* , *FeretAngle* and *minFeret* . 29 All parameters describing length or area are expressed in pixels or square pixels respectively.

#### Statistical Analysis

In this study, we employed two types of analysis of variance (ANOVA) to assess the data from the computerised image evaluation. Data from all verum and SNC experiments were analysed using a two-way ANOVA (CoStat, v. 6.311, CoHort Software, Monterey, United States) with α set at 0.05 and with *patient* and *treatment* as independent factors. Additionally, data from each patient were analysed by a one-way ANOVA with *treatment* as the factor for green colour channel images.

Global significance was determined with F-tests. Pairwise mean comparison was performed two-tailed with the protected Fisher's least significant difference test (pairwise comparisons were evaluated only if the global F-test was significant, *p* < 0.05). The procedure gives a good safeguard against errors of type I and II and thus balances well between false-positive and false-negative conclusions. 30 31

### Experimental Series II

#### Blood Sample Collection

For the second series of experiments, 16 EDTA blood samples, each from a different patient, scheduled to be discarded after laboratory tests at the hospital in Arlesheim (Klinik Arlesheim, Switzerland), were used. The samples were selected and coded by a person not involved in the experiments. The selection involved checking in the hospital databank whether the patient had signed an informed consent form and whether the blood count and clinical chemistry results relevant for the present study were measured during the laboratory tests and were available. All blood tests were ordered by physicians not involved in the present study solely for diagnostic purposes. The samples were transported to the laboratories of the Society for Cancer Research (Arlesheim, Switzerland) and stored at −21°C until analysis. The anonymised blood analysis results were given to the researchers.

##### The Kaelin Blood Test

This consisted of the same procedures as described in experimental series I, with the exception that in both phases, no treatments were applied.

#### Scanning of the Chromatograms and Image Evaluation

With regard to the scanning of the chromatograms and image evaluation, the experimental series II included the same steps as described in experimental series I.

#### Statistical Analysis

Mean values of the pattern evaluation parameters of each blood sample were calculated in Excel. Correlations between the pattern evaluation parameters and blood parameters were determined by the Bravais–Pearson linear coefficient of correlation *r* (Cov(X,Y))/(SD(X)SD(Y)).

## Results

### Investigation of the Impact of Homeopathic Remedies on Blood *In Vitro* (Experimental Series I)

#### Study Sample

Table 1 describes the 13 donors (2 males and 11 females) recruited among the patients of the homeopathic physician (J.L.). The median age of the donors was 57 years (interquartile range [IQR]: 45–66 years). The diagnosed disorders varied and included, among others, injuries and ailments from the digestive, musculoskeletal, cardiovascular and respiratory systems. All conditions were classified according to the 10th revision of the International Statistical Classification of Diseases and Related Health Problems (ICD-10). 32 All patients have had prior homeopathic treatment, but most had not used it for their current disorder. Besides the pre-determined remedies *Pulsatilla* and *Natrium muriaticum* at 30c and 200c and placebo, the following individually prescribed homeopathic remedies were tested in the present study: *Nux vomica* 200c, *Symphytum officinale* 200c, *Arnica montana* 30c, *Gelsemium sempervirens* 30c, *Sulphur* 200c and 30c, *Hepar sulphur* 30c, *Ipecacuanha* 30c, *Lycopodium clavatum* 30c and *Phosphorus* 30c.

#### Visual Pattern Assessment

As shown in Fig. 2 , the patterns obtained in the Kaelin blood test display distinct features arising from the stationary and mobile phases. The lower part of the pattern shows a reddish residuum remaining after the stationary phase, followed by a faint trace of the mobile phase, and finally, a dark red, polymorphous form (target form) of accumulated blood pigments at the top. A borderline separates the residuum from the mobile phase trace. Vertical channels are visible on both the residuum and the mobile phase trace. Whilst the residuum and the trace of the mobile phase were similar throughout the whole pattern database, the target forms showed the highest degree of differentiation. Visually, patterns obtained from samples collected from different patients were readily distinguishable; however, differences between the coded treatment groups (individually prescribed treatment, *Pulsatilla* , *Natrium muriaticum* and placebo) were not readily apparent.

#### Computerised Image Evaluation

The computerised image evaluation focused on the ROIs containing the target forms (ROIs, see also Fig. 1S ). Table 2 presents *p* -values of two-way ANOVAs with *patient* and *treatment* as independent factors for the particle analysis run on binary ROIs of all patterns from 13 verum experiments (EDTA blood samples treated with individually prescribed remedies, *Pulsatilla* , *Natrium muriaticum* and placebo) and 13 SNC experiments (SNCs; EDTA blood samples treated with placebo). *p* -Values are presented for both RGB images and images separated into single-colour channels (red, green and blue). Full data are available in Supplementary file 2 (available in online version only). The pattern evaluation parameters analysed describe the particles' following characteristics: *count* , *average* size, *perimeter* , *minor* and *major* axes (the primary and secondary-axis of the best fitting ellipse), *circularity* (a value of 1.0 indicates a perfect circle; as the value approaches 0.0, it indicates an increasingly elongated shape), *solidity* (ratio of area to convex area), *Feret's* *diameter* (the longest distance between any two points along the selection boundary, also known as maximum caliper), *FeretAngle* (the angle between 0 and 180 degrees of the *Feret's diameter* ) and *minFeret* (minimum caliper diameter). All parameters describing length or area are expressed in pixels or square pixels respectively.

**Table 2:** *p* -Values from two-way analyses of variance with *patient* and *treatment* as independent factors for the analysis run on the binary regions of interest containing the target forms of patterns from verum and systematic negative control experiments

	Evaluation parameters	Verum experiments			Systematic negative control experiments		
		Patient	Treatment	Interaction	Patient	Treatment	Interaction
RGB	Count	** <0.0001 c**	0.8179	0.2776	** <0.0001 c**	0.6493	0.1016
	Average Size	** 0.0015 b**	0.2122	** 0.0015 b**	** <0.0001 c**	0.5432	0.2118
	Perimeter	** <0.0001 c**	0.1718	** 0.0004 c**	** <0.0001 c**	0.5721	0.2679
	Major	** <0.0001 c**	0.0851	** 0.0011 b**	** <0.0001 c**	0.7434	0.1710
	Minor	** <0.0001 c**	0.2165	** 0.0216 a**	** <0.0001 c**	0.9107	0.4331
	Circularity	** <0.0001 c**	** 0.0114 a**	** 0.0037 b**	** 0.0003 c**	0.8937	0.6386
	Solidity	** <0.0001 c**	** 0.0222 a**	** 0.0087 b**	** <0.0001 c**	0.9404	0.7506
	Feret	** <0.0001 c**	0.0756	** 0.0004 c**	** <0.0001 c**	0.8224	0.1578
	FeretAngle	0.0851	0.0771	0.4081	0.2412	0.3976	0.6496
	MinFeret	** <0.0001 c**	0.1217	** 0.0016 b**	** <0.0001 c**	0.8841	0.1965
Red	Count	** <0.0001 c**	0.3238	0.4981	** <0.0001 c**	0.0620	0.1756
	Average size	** <0.0001 c**	0.1270	0.1427	** <0.0001 c**	0.3114	0.3452
	Perimeter	** <0.0001 c**	0.1266	0.1524	** <0.0001 c**	0.2080	0.3905
	Major	** <0.0001 c**	0.4412	0.2185	** <0.0001 c**	0.2800	0.2985
	Minor	** <0.0001 c**	0.2044	0.1859	** <0.0001 c**	0.7432	0.1840
	Circularity	** <0.0001 c**	0.0596	0.2960	** <0.0001 c**	0.4535	0.9904
	Solidity	** <0.0001 c**	0.0627	0.5322	** <0.0001 c**	0.7534	0.8969
	Feret	** <0.0001 c**	0.2790	0.1489	** <0.0001 c**	0.2894	0.1698
	FeretAngle	** 0.0048 b**	0.7727	0.7850	0.7566	0.9239	** 0.0265 a**
	MinFeret	** 0.0001 c**	0.2484	0.2112	** <0.0001 c**	0.6857	0.1746
Green	Count	** <0.0001 c**	0.5589	** 0.0286 a**	** <0.0001 c**	0.9220	0.1640
	Average size	** 0.0019 b**	0.4800	** 0.0002 c**	** <0.0001 c**	0.6709	0.1242
	Perimeter	** <0.0001 c**	0.2997	** <0.0001 c**	** <0.0001 c**	0.6667	0.2035
	Major	** <0.0001 c**	0.1399	** <0.0001 c**	** <0.0001 c**	0.7591	0.1726
	Minor	** <0.0001 c**	0.4384	** 0.0077 b**	** <0.0001 c**	0.7505	0.4033
	Circularity	** <0.0001 c**	** 0.0271 a**	** 0.0006 c**	** <0.0001 c**	0.4053	0.3929
	Solidity	** <0.0001 c**	** 0.0204 a**	** 0.0008 c**	** <0.0001 c**	0.6677	0.1518
	Feret	** <0.0001 c**	0.1169	** <0.0001 c**	** <0.0001 c**	0.7226	0.1884
	FeretAngle	** 0.0058 b**	** 0.0322 a**	** 0.0191 a**	0.2495	0.7923	0.9276
	MinFeret	** 0.0042 b**	0.2346	** 0.0003 c**	** <0.0001 c**	0.6738	0.1702
Blue	Count	** <0.0001 c**	0.2609	** 0.0178 a**	** <0.0001 c**	** 0.0278 a**	0.4661
	Average size	** 0.0103 a**	0.4658	0.1867	** <0.0001 c**	0.5678	0.3838
	Perimeter	** <0.0001 c**	0.4363	0.0914	** <0.0001 c**	0.4994	0.4173
	Major	** <0.0001 c**	0.2674	0.1339	** <0.0001 c**	0.5690	0.2280
	Minor	0.0675	0.4912	** 0.0203 a**	** <0.0001 c**	0.5526	0.1658
	Circularity	** <0.0001 c**	0.2497	0.2330	** <0.0001 c**	0.3284	0.1708
	Solidity	** <0.0001 c**	0.2351	0.0686	** <0.0001 c**	0.5028	** 0.0416 a**
	Feret	** <0.0001 c**	0.3079	0.0560	** <0.0001 c**	0.5581	0.3380
	FeretAngle	** 0.0115 a**	** 0.0453 a**	0.0991	** 0.0314 a**	0.0648	0.6862
	MinFeret	** 0.0030 b**	0.4810	** 0.0046 b**	** <0.0001 c**	0.6299	0.1863

Notes: The values are presented for RGB images and separate colour channels (red, green and blue). Significances are marked in bold ( *p* < 0.05).

a *p* < 0.05.

b *p* < 0.01.

c *p* < 0.001.

Regarding the verum experiments, the factor *patient* was highly significant for RGB images ( *p* -values ranging from 0.0015 to <0.0001) for most analysed pattern evaluation parameters (9/10), indicating that blood samples from different patients produced distinct chromatographic patterns. The factor *treatment* was significant for 2/10 parameters ( *p* -values of 0.0114 and 0.0222), indicating that for these parameters there were statistically significant differences between the treatment groups. Finally, the interaction between *patient* and *treatment* showed statistical significance for 8/10 parameters ( *p* -values ranging from 0.0216 to 0.0004), suggesting that the effects of the treatments varied strongly depending on the individual blood samples.

Analyzing images in separate single-colour channels revealed even greater significances for the green channel. Here, the factor *patient* was significant for all 10 parameters ( *p* -values ranging from 0.0058 to <0.0001). The factor *treatment* was significant for 3/10 parameters ( *p* -values from 0.0322 to 0.0204). Additionally, the interaction between *patient* and *treatment* remained significant for all 10 parameters ( *p* -values from 0.0286 to <0.0001). Analyses of red and blue channel images yielded less significant *p* -values.

The SNC experiments showed no significance for the *treatment* factor and the interaction between *patient* and *treatment* factors in RGB and green-channel images, whereas, as in the case of the verum experiments, high significances were found for the factor *patient* . Few low significances were found in the red and blue channel for the *treatment* factor (blue channel, *p* = 0.0278) and the interaction between *patient* and *treatment* factors (red channel, *p* = 0.0265; blue channel, *p* = 0.0416). Thus, the experimental set-up was completely stable regarding the factor *treatment* for RGB and green-channel images.

#### Computerised Evaluation of Images Obtained from Individual Blood Samples

The data evaluation of images obtained from individual blood samples are presented only for the ROIs of the green colour channel. As shown in Table 3 , 6 out of 13 tested blood samples showed significant differences between at least two treatments for at least one pattern evaluation parameter. In all six responding samples, three to one tested homeopathic treatments differed significantly from placebo; specifically, the individually prescribed treatment differed from placebo for 4/6 responding samples, *Pulsatilla* for 3/6 responding samples, and *Natrium muriaticum* for 5/6 responding samples. Regarding differences between the three tested homeopathic treatments, there were significant differences between the individually prescribed treatment and *Pulsatilla* for 2/6 responding samples, and between *Pulsatilla* and *Natrium muriaticum* for 3/6 responding samples. The F-values of the significances are shown in Fig. 3A , whereas those of corresponding negative control experiments are shown in Fig. 3B . Finally, Fig. 4 depicts the differences in the mean values of the best differentiating pattern evaluation parameters between the four treatments for all responding samples (R2, R6, R8, R9, R11, R12).

**Table 3:** F-test results from one-way analyses of variance for the factor *treatment* of the image analyses run on regions of interest containing the target form of the green colour channel of blood samples responding to the treatments ( *p* < 0.05)

			Blood sample																	
PEP	Treatment	*n*	R2			R6			R8			R9			R11			R12		
			F	*p*	Mean	F	*p*	Mean	F	*p*	Mean	F	*p*	Mean	F	*p*	Mean	F	*p*	Mean
Count	Individual	9				4.05	0.0151 a	10.33 ab							3.68	0.0221 a	11.56 c			
	*Pulsatilla*	9						12.67 a									15.67 ab			
	*Nat-mur.*	9						9.89 ab									12.22 bc			
	Placebo	9						7.89 b									16.22 a			
Average size	Individual	9	3.64	0.0230 a	19.05 × 10 ^3^ b	3.64	0.0229 a	18.06 × 10 ^3^ b												
	*Pulsatilla*	9			14.55 × 10 ^3^ b			15.26 × 10 ^3^ b												
	*Nat-mur.*	9			23.53 × 10 ^3^ b			19.05 × 10 ^3^ ab												
	Placebo	9			47.37 × 10 ^3^ a			23.22 × 10 ^3^ a												
Perimeter	Individual	9	3.65	0.0227 a	18.01 × 10 ^2^ b	3.87	0.0181 a	12.10 × 10 ^2^ b												
	*Pulsatilla*	9			13.43 × 10 ^2^ b			10.68 × 10 ^2^ b												
	*Nat-mur.*	9			22.67 × 10 ^2^ b			13.16 × 10 ^2^ ab												
	Placebo	9			45.81 × 10 ^2^ a			16.63 × 10 ^2^ a												
Major	Individual	9	3.65	0.0226 a	175.62 b															
	*Pulsatilla*	9			129.94 b															
	*Nat-mur.*	9			221.08 b															
	Placebo	9			430.44 a															
Minor	Individual	9	4.46	0.0100 a	40.83 b	5.29	0.0045 b	67.24 b	3.05	0.0424 a	51.37 ab									
	*Pulsatilla*	9			40.76 b			57.29 b			69.21 a									
	*Nat-mur.*	9			46.35 b			67.21 b			50.59 ab									
	Placebo	9			84.79 a			84.96 a			40.91 b									
Circularity	Individual	9	5.56	0.0035 b	0.64 a							3.75	0.0205 a	0.59 ab				3.36	0.0307 a	0.59 ab
	*Pulsatilla*	9			0.61 a									0.49 b						0.67 a
	*Nat-mur.*	9			0.57 a									0.67 a						0.51 b
	Placebo	9			0.34 b									0.58 ab						0.62 a
Solidity	Individual	9	4.26	0.0122 a	0.68 a				2.92	0.0490 a	0.71 ab	4.07	0.0147 a	0.70 ab						
	*Pulsatilla*	9			0.67 a						0.65 b			0.65 b						
	*Nat-mur.*	9			0.65 a						0.70 ab			0.75 a						
	Placebo	9			0.50 b						0.74 a			0.68 b						
Feret	Individual	9	3.87	0.0181 a	277.90 b	3.64	0.0230 a	209.67 b												
	*Pulsatilla*	9			210.93 b			194.60 b												
	*Nat-mur.*	9			357.29 b			223.76 ab												
	Placebo	9			707.74 a			278.67 a												
Feret Angle	Individual	9							4.29	0.0119 a	105.07 a									
	*Pulsatilla*	9									79.19 b									
	*Nat-mur.*	9									93.69 ab									
	Placebo	9									89.49 b									
Min Feret	Individual	9	4.89	0.0066 b	91.61 b	7.47	0.0006 c	113.06 b	3.16	0.0378 a	102.08 ab									
	*Pulsatilla*	9			89.09 b			99.81 b			137.11 a									
	*Nat-mur.*	9			106.16 b			117.39 b			94.81 b									
	Placebo	9			210.86 a			155.45 a			81.33 b									

Abbreviation: PEP, pattern evaluation parameter.

a *p* < 0.05

b *p* < 0.01

c *p* < 0.001.

**Fig. 3:**
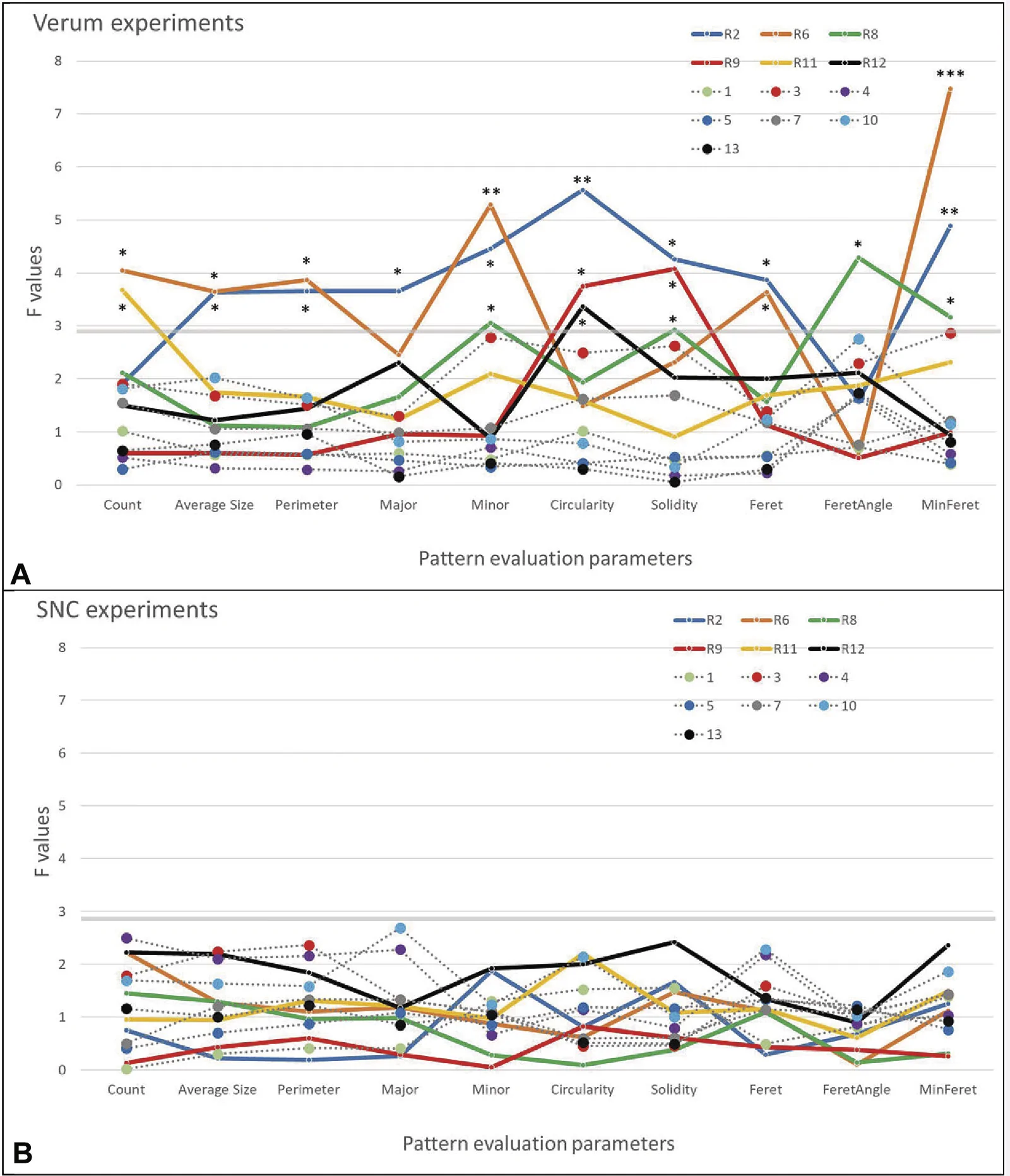
*F* -values from one-way analyses of variance of the particle analyses with the factor *treatment* for verum ( **A** ) and systematic negative control experiments ( **B** ). The particle analyses were performed on ROIs of the green colour channel of the RGB colour space. Blood samples responding to treatments in verum experiments (R-samples) are marked by coloured plot lines. Marker points above the grey line ( *F* -value ca. 2.90, *p* < 0.05) indicate statistically significant differences between the treatments for a given pattern evaluation parameter. * *p* < 0.05; ** *p* < 0.01; *** *p* < 0.001.

**Fig. 4:**
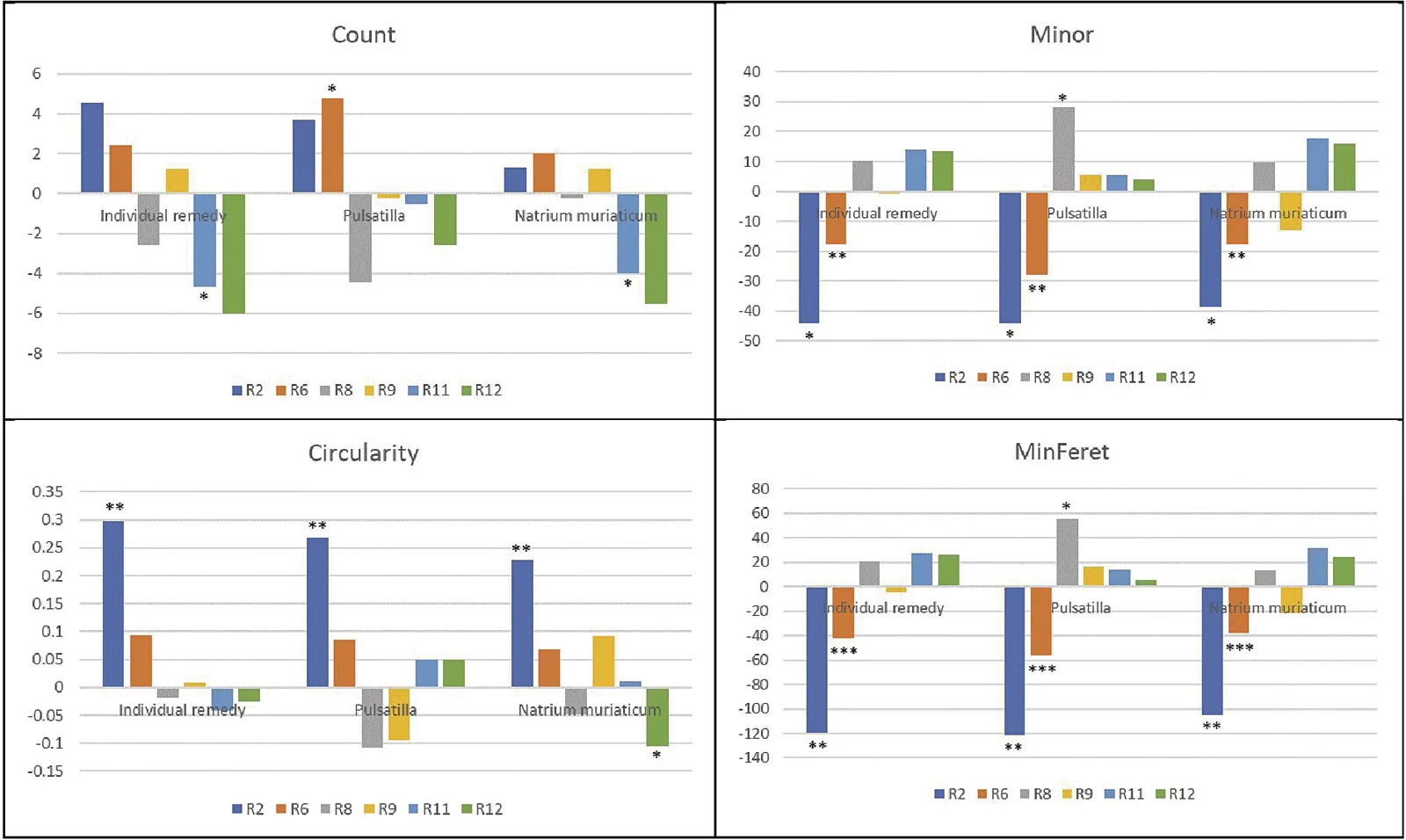
The four charts depict the differences between homeopathic treatments (individual treatment, *Pulsatilla* and *Natrium muriaticum* ) compared with a placebo, for four pattern evaluation parameters: count, minor, circularity and minFeret. Statistical significances between the given treatment and placebo, based on a one-way ANOVA conducted for the data on pattern evaluation parameters and individual blood samples ( Table 3 ), are indicated by asterisks. * *p* < 0.05; ** *p* < 0.01; *** *p* < 0.001; R2, R6, R8, R9, R11, R12: blood samples responding to the treatments. ANOVA, analysis of variance.

#### Responders within the Study Sample

The majority of responding blood samples (5/6) were derived from donors with acute conditions, whilst most donors who donated the non-responding samples (5/7) suffered from chronic diseases. The median age of donors of responding samples was 58 years (IQR: 57–67.25 years) compared with 51 years (IQR: 34–60 years) for donors of non-responding samples. Among the donors who provided blood samples that yielded a response, none was undergoing homeopathic treatment for the current condition, whereas 2/7 donors in the non-responding group had been undergoing treatment with homeopathy for the current condition. With regard to the potency levels of the homeopathic remedies, both the responding and non-responding samples included donors who had been prescribed remedies at both 30c and 200c potency levels ( Table 1 ), and no clear pattern was observed between potency level and response. Due to the small number of donors ( *n* = 13), a statistical evaluation of the above-mentioned parameters potentially responsible for the response to the homeopathic treatment was not performed, and we consider these observations too limited to draw any conclusions about broader trends.

### Correlations between Blood Parameters and Pattern Evaluation Parameters (Experimental Series II)

The pattern evaluation parameters for ROIs of green channel images were tested for correlations with the blood parameters. Table 4 shows strong correlations ( *r* ≥ 0.70 or *r* ≤ −0.70), whereas full data are available in Supplementary file 2 (available in online version only). The strongest correlations involved parameters related to red blood cells (haemoglobin, haematocrit, mean corpuscular volume [MCV], mean corpuscular haemoglobin [MCH], erythrocytes, red cell distribution width) and liver function (aspartate aminotransferase [AST], γ-glutamyl transferase [GGT], lactate dehydrogenase [LDH]). The scatter plots in Fig. 5 show the intersection points of haemoglobin values with several pattern evaluation parameters that strongly correlate with haemoglobin, underlining the robustness of the here-reported correlations.

**Table 4:** Pearson's *r* -values of correlations between the blood parameters (count and clinical chemistry) and pattern evaluation parameters for regions of interest containing the target forms of green channel images

	Count	Average size	Perimeter	Major	Minor	Circularity	Solidity	Feret	Feret angle	MinFeret
Haemoglobin	**0.91**	**−0.84**	**−0.83**	**−0.84**	−0.68	0.60	0.68	**−0.83**	0.65	**−0.76**
Haematocrit	**0.92**	**−0.88**	**−0.88**	**−0.88**	**−0.77**	0.69	**0.77**	**−0.88**	**0.70**	**−0.84**
MCV	−0.59	0.67	**0.72**	**0.71**	0.55	−0.59	−0.67	**0.73**	**−0.72**	0.68
MCH	−0.50	0.65	**0.70**	0.69	0.58	−0.65	**−0.73**	**0.71**	**−0.74**	0.70
Erythrocytes	**0.86**	**−0.84**	**−0.85**	**−0.85**	**−0.71**	0.67	**0.76**	**−0.86**	**0.73**	**−0.81**
RDW %	**−0.72**	0.69	**0.71**	**0.71**	0.48	−0.41	−0.53	**0.71**	−0.60	0.60
AST	−0.67	**0.85**	**0.86**	**0.84**	0.43	−0.45	−0.64	**0.84**	**−0.78**	0.64
GGT	−0.42	0.59	0.67	0.61	0.13	−0.32	−0.57	0.64	**−0.74**	0.43
LDH	−0.68	**0.70**	0.68	**0.73**	**0.74**	**−0.74**	−0.67	**0.71**	−0.51	**0.73**

Abbreviations: AST, aspartate aminotransferase; GGT, γ -glutamyl transferase; LDH, lactate dehydrogenase; MCH, mean corpuscular haemoglobin; MCV, mean corpuscular volume; RDW %, red cell distribution width.

Note: Strong correlations ( *r* ≥ 0.70 or *r* ≤ −0.70) are marked in bold.

**Fig. 5:**
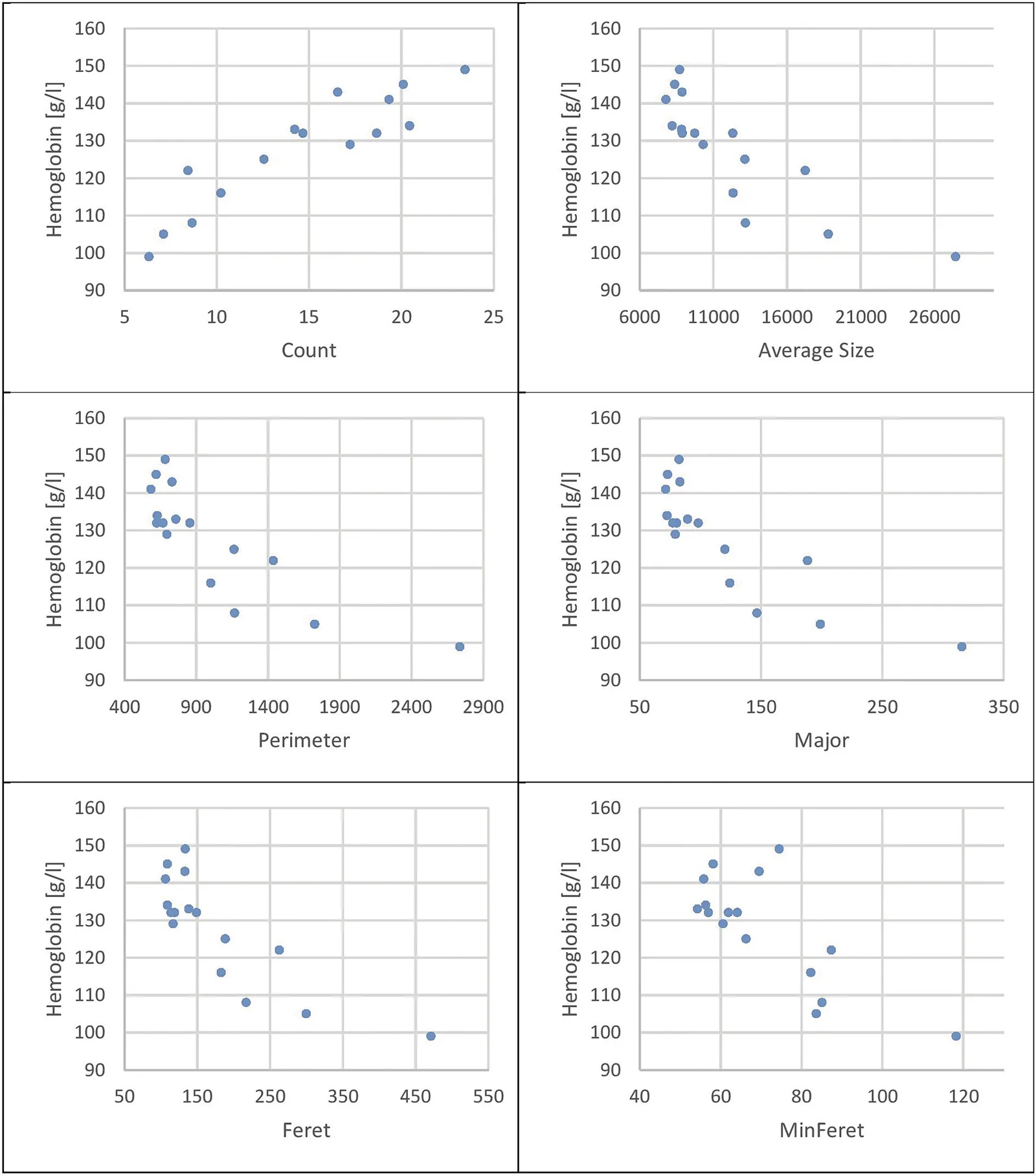
Scatter plots showing correlations between haemoglobin [g/L] and mean values of pattern evaluation parameters: count, average size, perimeter, major, Feret and minFeret for 16 blood samples.

## Discussion

In the verum experiments of the present study, each of the 13 blood samples collected from different patients was treated *in vitro* with an individually prescribed treatment, *Pulsatilla, Natrium muriaticum* , at either 30c or 200c potency, and placebo. For the 13 patients 9 different individual remedies were prescribed, derived from six substances of plant and three of mineral origin, including one remedy prescribed at both 30c and 200c potency level ( Table 1 ). Including the pre-determined remedies ( *Pulsatilla* , *Natrium muriaticum* ), the study tested 11 homeopathic remedies derived from seven substances of plant and four of mineral origin, with three remedies included at both potency levels (i.e., *Pulsatilla* , *Natrium muriaticum* and *Sulphur* ). The analysis performed using the Kaelin blood test (ascending paper chromatography) showed that the response of the blood samples toward the treatments, both individually prescribed as well as pre-determined, varied from patient to patient, and thus was individual.

The pattern evaluation focused on the dark, polymorphic shape visible in the upper part of each chromatogram (target form), which was the most differentiated and the darkest feature within the pattern, indicating that most blood pigments were concentrated there.

The analysis of the data from particle analysis of ROIs containing the target forms using a two-way ANOVA with *patient* and *treatment* as independent factors revealed statistically significant effects for both factors. However, the significances for the factor *treatment* were rather weak. The high significances of the factor *patient* were unsurprising due to inherent variations in blood composition among donors. Interestingly, the interaction between the factors *patient* and *treatment* was also highly significant for most pattern evaluation parameters. This finding suggests that the effects of the *in vitro* treatments were different in different blood samples, and thus were patient-specific. These effects were even more pronounced when analysing only the green channel of the RGB images.

The experimental system stability, as evaluated through SNC experiments, was robust. Patterns from placebo-treated samples, analysed following the same randomisation design as the verum experiment, did not show any statistical differences for RGB and green channel images. This means that—with a very high probability—the results obtained in the verum experiments were due to the homeopathic treatment and not due to any systematic or stochastic errors in the experimental set-up.

The one-way ANOVAs run on data from the 13 single blood samples showed that the applied pattern evaluation approach identified six blood samples that responded to the treatments. It was remarkable that five out of these six responding blood samples were derived from donors with acute disorders. The majority (5/7) of donors of the non-responding samples suffered from chronic diseases. It could also be observed that donors whose blood samples responded to the *in vitro* treatments were older (median age: 58 years; IQR: 57–67.25 years) than those whose samples did not respond (median age: 51 years; IQR: 34–60 years). However, due to the small and highly heterogeneous study sample, no firm general conclusion can be drawn based on this responder analysis. Additionally, as the study included only patients who had previously undergone homeopathic treatment (although with remedies different from those tested *in vitro* ), we acknowledge that residual effects of prior remedies cannot be fully ruled out, despite efforts to minimise direct overlap. In future studies, incorporating a washout period might enhance the responsiveness of blood samples.

The self-organisation of the blood haemolysates into patterns during both phases of the Kaelin blood test is driven by capillary forces acting as the liquids soak into the chromatographic papers. In patterns obtained in the same environmental conditions, the emerged forms depend on the blood composition and characteristics. It can be assumed that the target form, being the darkest feature within the pattern, is formed by the greatest cumulation of the blood pigment haemoglobin and its degradation products. Indeed, strong positive correlations between the particle count—calculated from ROIs on green channel images—and red blood cell parameters (haemoglobin, haematocrit, and erythrocyte count; *r* = 0.91, 0.92, and 0.86, respectively) indicate that samples with higher erythrocyte and haemoglobin content produced substantially more detectable particles within the patterns. Conversely, haemoglobin, haematocrit and erythrocyte count displayed strong negative correlations with *average size* , *perimeter* and *major axis* ( *r* between –0.71 and –0.88), suggesting that in samples richer in red cell-derived material and higher erythrocyte count, the pigment was distributed into a larger number of smaller structures rather than into a few larger aggregates. In contrast, MCV and MCH showed the opposite profile: both parameters correlated negatively with *particle count* ( *r* = –0.59 and –0.50) but positively with size-related parameters such as *average size* , *perimeter* , *major axis* and *minFeret* ( *r* = 0.55–0.73), indicating that samples containing larger erythrocytes tended to form fewer but larger pattern structures. Furthermore, the liver-related enzymes (AST, GGT, LDH) demonstrated moderate to strong positive correlations with size parameters ( *r* up to 0.86) and negative correlations with *particle count* ( *r* between –0.42 and –0.68), suggesting that non-haematological blood characteristics also contributed to the observed variability in pattern formation.

In context of the *in vitro* homeopathic treatments applied to the samples in experimental series I, the correlations detected may suggest that the tested homeopathic remedies primarily influenced the final distribution of haemoglobin, its degradation products and liver function-related blood components on the chromatographic paper. However, the mechanisms through which this effect occurred remain unclear.

For the purpose of the present study, the protocol of the original Kaelin blood test 24 25 was modified. Some modifications, such as adjusting blood haemolysate concentration and covering the chromatographic papers with beakers, aimed to eliminate any experimenter interactions that might have introduced bias. Other modifications, such as adding globules of the tested homeopathic remedy to both the stationary and mobile phases and setting fixed incubation times, adapted the former diagnostic test for the intended scope of analyzing the influence of the homeopathic treatments.

For image evaluation, we performed particle analyses of ROIs containing the target forms in RGB images and separate red, green and blue channel images. To define the ROIs consistently across the database, all images were scaled to the same height. This approach ensured consistent positioning of the target forms and facilitated the designation of identical ROIs for all images, regardless of the original pattern height. However, it is important to acknowledge that the scaling, followed by bi-cubic interpolation, might affect the characteristics of particles within the target forms. The validity of the results from the verum experiments is confirmed by non-significant results obtained in the corresponding SNC experiments.

Both the adjusted protocol of the Kaelin blood test and the image evaluation procedures used in the present study were developed *ad hoc* . Thus, the experimental procedures are not yet finalised and are open for further development and improvement. It should be examined whether, by implementing different procedures into the experimental protocol (e.g. other blood haemolysate concentrations, globules size, incubation times of the remedies) and pattern evaluation techniques (e.g. different scaling heights, interpolation procedures and alternative colour spaces), the test can be optimised.

With regard to basic homeopathy research, studies testing the influence of different homeopathic preparations on physical, 7 33 34 35 cellular, 36 37 38 39 plant 40 41 and animal models 39 42 43 44 have been conducted; as well as studies in veterinary homeopathy 45 46 47 and clinical trials. 48 49 50 51 There were also attempts to apply homeopathic treatments to human body fluids *in vitro* and to perform analysis using copper-chloride biocrystallisation 8 52 and capillary dynamolysis (ascending paper chromatography with added reagents). 21

The model presented in our study offers a promising tool for investigating the *in vitro* reactions of human blood samples to homeopathic preparations, providing insights into individualised biological responses. Whilst these findings are limited to laboratory settings, the model may serve as a foundation for future research exploring potential applications in treatment monitoring or adjustment, with the ultimate goal of supporting more personalised prescribing practices in homeopathy.

## Highlights

- Blood samples from 13 patients were treated *in vitro* with homeopathic preparations.
- A chromatographic test was employed to obtain patterns from the treated blood samples.
- Pattern evaluation was conducted with the ImageJ software.
- Blood samples showed significant differences in response to the various treatments.
- Responses varied by donor, indicating the individual nature of reactions.
