# Supplementary material for: A Chromatographic Test Reveals Homeopathic Preparations Affect Human Blood In Vitro
Source: Homeopathy. 2026 May 13;115(4):242–57. doi: 10.1055/a-2748-6796 (PMC13716517; doi:10.1055/a-2748-6796)
Supplement: Supplementary file 1 [file 10-1055-a-2748-6796_28726672.pdf]

Supplementary File 1. Video recording of the Kaelin test experimental procedure

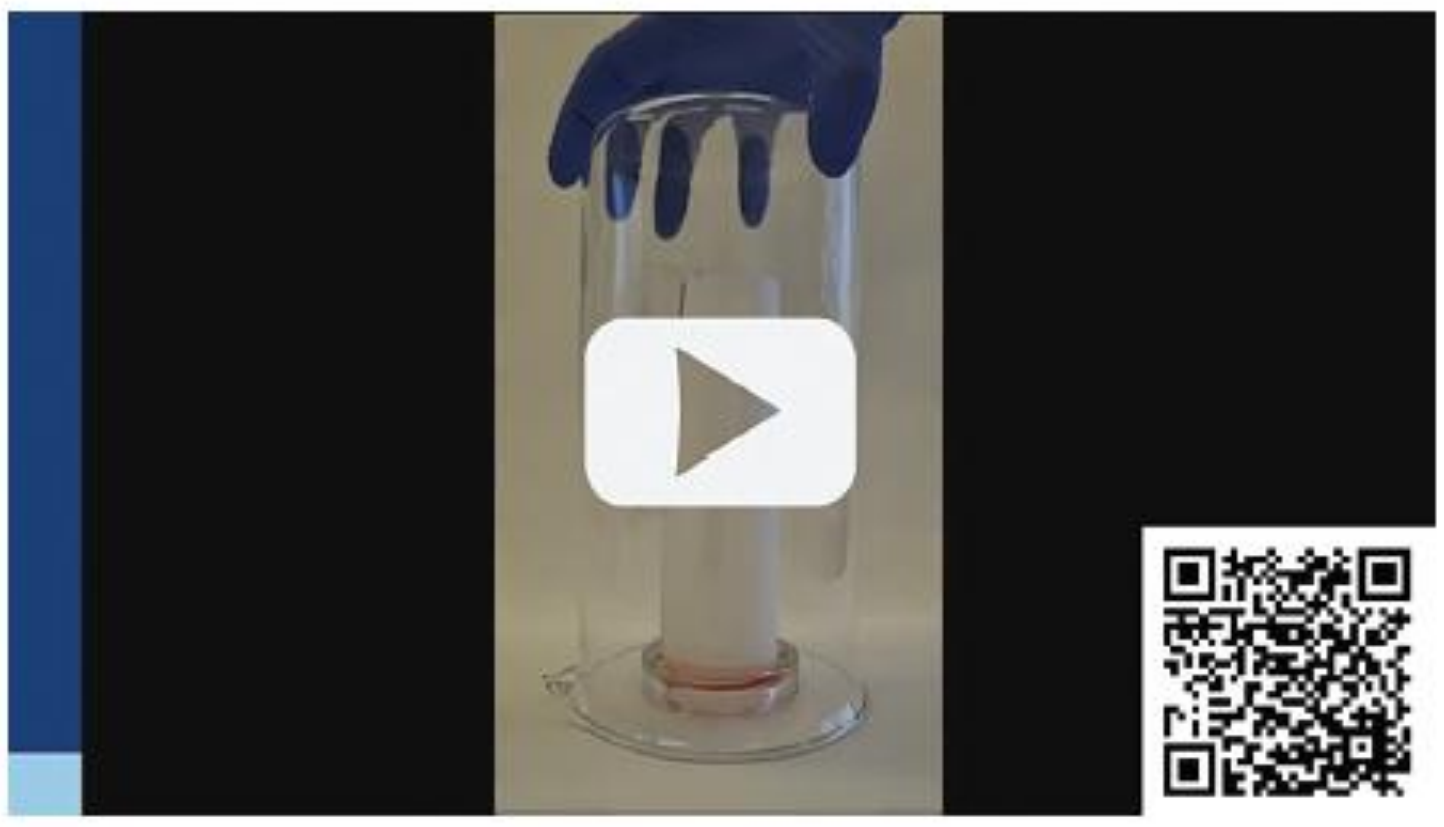

Available at: https://www.thieme.de/de/q.htm?p=opn/cs/26/1/27942644-95502307

Supplementary File 2

Table S1: F-test results of the two-way ANOVA (analysis of variance) of all blood samples, with *patient* and *treatment* as independent factors of the particle analysis run on regions of interest containing the target form (ROI) marked in the RGB images converted to binary.
LEGEND: * – p < 0.05; ** – p < 0.01; *** – p < 0.001, ns – not significant.

Pattern eval. parameter	Factor	Verum		SNC		Treatment	Verum		SNC	
		F	*p*	F	*p*		n	Mean	n	Mean
Count	Patient	11.86	<0.0001 ***	16.64	<0.0001 ***	Individual	117	13.81 a	117	14.67 a
	Treatment	0.31	0.8179 ns	0.55	0.6493 ns	*Pulsatilla*	117	14.06 a	117	14.67 a
	Interaction	1.13	0.2776 ns	1.33	0.1016 ns	*Nat-mur.*	117	13.58 a	117	13.89 a
						Placebo	117	14.25 a	117	14.15 a
Average size	Patient	2.72	0.0015 **	4.75	<0.0001 ***	Individual	117	14.24 x 10^3 a	117	14.65 x 10^3 a
	Treatment	1.51	0.2122 ns	0.72	0.5432 ns	*Pulsatilla*	117	15.14 x 10^3 a	117	14.27 x 10^3 a
	Interaction	1.92	0.0015 **	1.19	0.2118 ns	*Nat-mur.*	117	14.21 x 10^3 a	117	15.18 x 10^3 a
						Placebo	117	16.26 x 10^3 a	117	15.52 x 10^3 a
Perimeter	Patient	6.53	<0.0001 ***	6.89	<0.0001 ***	Individual	117	10.63 x 10^2 a	117	10.71 x 10^2 a
	Treatment	1.67	0.1718 ns	0.67	0.5721 ns	*Pulsatilla*	117	11.48 x 10^2 a	117	10.44 x 10^2 a
	Interaction	2.06	0.0004 ***	1.14	0.2679 ns	*Nat-mur.*	117	10.57 x 10^2 a	117	10.88 x 10^2 a
						Placebo	117	12.53 x 10^2 a	117	11.45 x 10^2 a
Major	Patient	5.61	<0.0001 ***	5.88	<0.0001 ***	Individual	117	106.73 b	117	110.16 a
	Treatment	2.22	0.0851 ns	0.41	0.7434 ns	*Pulsatilla*	117	114.18 ab	117	108.95 a
	Interaction	1.96	0.0011 **	1.23	0.171 ns	*Nat-mur.*	117	107.81 b	117	114.01 a
						Placebo	117	128.36 a	117	115.24 a
Minor	Patient	5.10	<0.0001 ***	4.66	<0.0001 ***	Individual	117	41.97 a	117	44.13 a
	Treatment	1.49	0.2165 ns	0.18	0.9107 ns	*Pulsatilla*	117	43.12 a	117	43.06 a
	Interaction	1.57	0.0216 *	1.02	0.4331 ns	*Nat-mur.*	117	42.45 a	117	44.84 a
						Placebo	117	46.46 a	117	43.72 a
Circularity	Patient	5.05	<0.0001 ***	3.13	0.0003 ***	Individual	117	0.65 a	117	0.64 a
	Treatment	3.73	0.0114 *	0.20	0.8937 ns	*Pulsatilla*	117	0.65 a	117	0.64 a
	Interaction	1.80	0.0037 **	0.90	0.6386 ns	*Nat-mur.*	117	0.65 a	117	0.64 a
						Placebo	117	0.62 b	117	0.65 a
Solidity	Patient	7.54	<0.0001 ***	4.04	<0.0001 ***	Individual	117	0.73 a	117	0.72 a
	Treatment	3.23	0.0222 *	0.13	0.9404 ns	*Pulsatilla*	117	0.73 a	117	0.72 a
	Interaction	1.70	0.0087 **	0.83	0.7506 ns	*Nat-mur.*	117	0.73 a	117	0.73 a
						Placebo	117	0.71 b	117	0.73 a
Feret	Patient	6.48	<0.0001 ***	6.31	<0.0001 ***	Individual	117	165.8 b	117	170.08 a
	Treatment	2.31	0.0756 ns	0.30	0.8224 ns	*Pulsatilla*	117	177.74 ab	117	167.78 a
	Interaction	2.09	0.0004 ***	1.25	0.1578 ns	*Nat-mur.*	117	167.02 b	117	173.66 a
						Placebo	117	200.22 a	117	177.07 a
Feret Angle	Patient	1.61	0.0851 ns	1.26	0.2412 ns	Individual	117	97.67 a	117	97.16 a
	Treatment	2.30	0.0771 ns	0.99	0.3976 ns	*Pulsatilla*	117	96.49 ab	117	96.72 a
	Interaction	1.04	0.4081 ns	0.89	0.6496 ns	*Nat-mur.*	117	97.55 a	117	94.55 a
						Placebo	117	93.05 b	117	94.99 a
Min Feret	Patient	4.15	<0.0001 ***	5.44	<0.0001 ***	Individual	117	78.58 b	117	82.28 a
	Treatment	1.94	0.1217 ns	0.22	0.8841 ns	*Pulsatilla*	117	81.94 ab	117	78.93 a
	Interaction	1.91	0.0016 **	1.21	0.1965 ns	*Nat-mur.*	117	78.95 b	117	82.25 a
						Placebo	117	88.97 a	117	81.24 a

Table S2: F-test results of the two-way ANOVA (analysis of variance) of all blood samples, with *patient* and *treatment* as independent factors of the particle analysis run on regions of interest containing the target form (ROI) marked in the red colour channel converted to binary.
LEGEND: * – p < 0.05; ** – p < 0.01; *** – p < 0.001, ns – not significant.

Pattern eval. parameter	Factor	Verum		SNC		Treatment	Verum		SNC	
		F	*p*	F	*p*		n	Mean	n	Mean
Count	Patient	8.26	<0.0001 ***	14.56	<0.0001 ***	Individual	117	52.37 a	117	57.20 a
	Treatment	1.16	0.3238 ns	2.46	0.0620 ns	*Pulsatilla*	117	54.50 a	117	50.35 b
	Interaction	0.98	0.4981 ns	1.23	0.1756 ns	*Nat-mur.*	117	50.93 a	117	55.29 ab
						Placebo	117	55.48 a	117	54.52 ab
Average size	Patient	5.50	<0.0001 ***	9.58	<0.0001 ***	Individual	117	30.66 x 10^2 ab	117	29.34 x 10^2 a
	Treatment	1.91	0.1270 ns	1.19	0.3114 ns	*Pulsatilla*	117	30.00 x 10^2 ab	117	32.16 x 10^2 a
	Interaction	1.27	0.1427 ns	1.08	0.3452 ns	*Nat-mur.*	117	33.29 x 10^2 a	117	30.84 x 10^2 a
						Placebo	117	29.37 x 10^2 b	117	31.63 x 10^2 a
Perimeter	Patient	3.83	<0.0001 ***	9.25	<0.0001 ***	Individual	117	301.55 ab	117	285.74 a
	Treatment	1.91	0.1266 ns	1.52	0.2080 ns	*Pulsatilla*	117	296.67 ab	117	315.83 a
	Interaction	1.26	0.1524 ns	1.05	0.3905 ns	*Nat-mur.*	117	331.54 a	117	296.87 a
						Placebo	117	291.74 b	117	310.46 a
Major	Patient	6.45	<0.0001 ***	9.12	<0.0001 ***	Individual	117	30.59 a	117	29.96 a
	Treatment	0.90	0.4412 ns	1.28	0.2800 ns	*Pulsatilla*	117	30.74 a	117	32.62 a
	Interaction	1.19	0.2185 ns	1.12	0.2985 ns	*Nat-mur.*	117	32.98 a	117	30.94 a
						Placebo	117	30.87 a	117	31.55 a
Minor	Patient	7.50	<0.0001 ***	7.25	<0.0001 ***	Individual	117	12.41 ab	117	12.57 a
	Treatment	1.54	0.2044 ns	0.41	0.7432 ns	*Pulsatilla*	117	12.00 b	117	13.17 a
	Interaction	1.22	0.1859 ns	1.22	0.1840 ns	*Nat-mur.*	117	13.40 a	117	12.57 a
						Placebo	117	12.43 ab	117	12.74 a
Circularity	Patient	4.00	<0.0001 ***	4.48	<0.0001 ***	Individual	117	0.76 a	117	0.75 a
	Treatment	2.49	0.0596 ns	0.88	0.4535 ns	*Pulsatilla*	117	0.75 ab	117	0.74 a
	Interaction	1.12	0.2960 ns	0.52	0.9904 ns	*Nat-mur.*	117	0.75 b	117	0.75 a
						Placebo	117	0.75 b	117	0.75 a
Solidity	Patient	10.07	<0.0001 ***	8.63	<0.0001 ***	Individual	117	0.78 a	117	0.77 a
	Treatment	2.45	0.0627 ns	0.40	0.7534 ns	*Pulsatilla*	117	0.77 ab	117	0.77 a
	Interaction	0.96	0.5322 ns	0.71	0.8969 ns	*Nat-mur.*	117	0.77 b	117	0.78 a
						Placebo	117	0.77 b	117	0.77 a
Feret	Patient	5.34	<0.0001 ***	8.71	<0.0001 ***	Individual	117	48.28 a	117	47.12 a
	Treatment	1.29	0.2790 ns	1.26	0.2894 ns	*Pulsatilla*	117	48.00 a	117	51.18 a
	Interaction	1.26	0.1489 ns	1.24	0.1698 ns	*Nat-mur.*	117	52.58 a	117	48.03 a
						Placebo	117	48.24 a	117	49.51 a
Feret Angle	Patient	2.42	0.0048 **	0.70	0.7566 ns	Individual	117	98.16 a	117	97.69 a
	Treatment	0.37	0.7727 ns	0.16	0.9239 ns	*Pulsatilla*	117	97.81 a	117	97.49 a
	Interaction	0.80	0.7850 ns	1.54	0.0265 *	*Nat-mur.*	117	97.52 a	117	97.30 a
						Placebo	117	97.27 a	117	97.17 a
Min Feret	Patient	3.45	0.0001 ***	6.78	<0.0001 ***	Individual	117	23.35 ab	117	23.18 a
	Treatment	1.38	0.2484 ns	0.50	0.6857 ns	*Pulsatilla*	117	22.41 b	117	24.52 a
	Interaction	1.19	0.2112 ns	1.23	0.1746 ns	*Nat-mur.*	117	25.15 a	117	23.32 a
						Placebo	117	23.22 ab	117	23.75 a

Table S3: F-test results of the two-way ANOVA (analysis of variance) of all blood samples, with *patient* and *treatment* as independent factors of the particle analysis run on regions of interest containing the target form (ROI) marked in the green colour channel converted to binary.
LEGEND: * – p < 0.05; ** – p < 0.01; *** – p < 0.001, ns – not significant.

Pattern eval. parameter	Factor	Verum		SNC		Treatment	Verum		SNC	
		F	*p*	F	*p*		n	Mean	n	Mean
Count	Patient	12.23	<0.0001 ***	16.47	<0.0001 ***	Individual	117	11.00 a	117	12.05 a
	Treatment	0.69	0.5589 ns	0.16	0.9220 ns	*Pulsatilla*	117	11.72 a	117	11.66 a
	Interaction	1.53	0.0286 *	1.24	0.1640 ns	*Nat-mur.*	117	11.09 a	117	11.67 a
						Placebo	117	11.56 a	117	11.83 a
Average size	Patient	2.66	0.0019 **	5.92	<0.0001 ***	Individual	117	17.98×10^3 a	117	19.58×10^3 a
	Treatment	0.83	0.4800 ns	0.52	0.6709 ns	*Pulsatilla*	117	18.58×10^3 a	117	18.09×10^3 a
	Interaction	2.14	0.0002 ***	1.29	0.1242 ns	*Nat-mur.*	117	17.95×10^3 a	117	18.19×10^3 a
						Placebo	117	19.83×10^3 a	117	18.53×10^3 a
Perimeter	Patient	6.58	<0.0001 ***	7.39	<0.0001 ***	Individual	117	13.13×10^2 a	117	14.11×10^2 a
	Treatment	1.23	0.2997 ns	0.52	0.6667 ns	*Pulsatilla*	117	13.70×10^2 a	117	13.04×10^2 a
	Interaction	2.35	<0.0001 ***	1.20	0.2035 ns	*Nat-mur.*	117	13.15×10^2 a	117	12.86×10^2 a
						Placebo	117	15.11×10^2 a	117	13.43×10^2 a
Major	Patient	6.35	<0.0001 ***	6.55	<0.0001 ***	Individual	117	132.55 b	117	144.56 a
	Treatment	1.84	0.1399 ns	0.39	0.7591 ns	*Pulsatilla*	117	138.22 ab	117	136.63 a
	Interaction	2.34	<0.0001 ***	1.23	0.1726 ns	*Nat-mur.*	117	135.75 ab	117	135.74 a
						Placebo	117	155.43 a	117	136.53 a
Minor	Patient	3.69	<0.0001 ***	5.55	<0.0001 ***	Individual	117	51.93 a	117	56.22 a
	Treatment	0.91	0.4384 ns	0.40	0.7505 ns	*Pulsatilla*	117	54.26 a	117	54.59 a
	Interaction	1.71	0.0077 **	1.04	0.4033 ns	*Nat-mur.*	117	52.43 a	117	54.13 a
						Placebo	117	56.52 a	117	52.78 a
Circularity	Patient	4.49	<0.0001 ***	5.40	<0.0001 ***	Individual	117	0.61 a	117	0.58 a
	Treatment	3.09	0.0271 *	0.97	0.4053 ns	*Pulsatilla*	117	0.59 ab	117	0.58 a
	Interaction	2.02	0.0006 ***	1.05	0.3929 ns	*Nat-mur.*	117	0.60 a	117	0.60 a
						Placebo	117	0.56 b	117	0.60 a
Solidity	Patient	7.09	<0.0001 ***	8.57	<0.0001 ***	Individual	117	0.71 a	117	0.70 a
	Treatment	3.30	0.0204 *	0.52	0.6677 ns	*Pulsatilla*	117	0.70 ab	117	0.70 a
	Interaction	2.00	0.0008 ***	1.26	0.1518 ns	*Nat-mur.*	117	0.71 a	117	0.70 a
						Placebo	117	0.68 b	117	0.71 a
Feret	Patient	7.09	<0.0001 ***	7.26	<0.0001 ***	Individual	117	205.32 b	117	222.87 a
	Treatment	1.98	0.1169 ns	0.44	0.7226 ns	*Pulsatilla*	117	215.32 ab	117	211.01 a
	Interaction	2.53	<0.0001 ***	1.22	0.1884 ns	*Nat-mur.*	117	210.27 ab	117	207.24 a
						Placebo	117	243.21 a	117	210.23 a
Feret Angle	Patient	2.37	0.0058 **	1.25	0.2495 ns	Individual	117	98.20 a	117	95.18 a
	Treatment	2.96	0.0322 *	0.35	0.7923 ns	*Pulsatilla*	117	95.56 ab	117	93.77 a
	Interaction	1.59	0.0191 *	0.67	0.9276 ns	*Nat-mur.*	117	94.27 ab	117	93.31 a
						Placebo	117	91.72 b	117	94.76 a
Min Feret	Patient	2.46	0.0042 **	6.96	<0.0001 ***	Individual	117	97.04 a	117	105.33 a
	Treatment	1.43	0.2346 ns	0.51	0.6738 ns	*Pulsatilla*	117	102.76 a	117	100.22 a
	Interaction	2.10	0.0003 ***	1.24	0.1702 ns	*Nat-mur.*	117	97.61 a	117	99.62 a
						Placebo	117	108.72 a	117	98.11 a

Table S4: F-test results of the two-way ANOVA (analysis of variance) of all blood samples, with *patient* and *treatment* as independent factors of the particle analysis run on regions of interest containing the target form (ROI) marked in the blue colour channel converted to binary. LEGEND: * – p < 0.05; ** – p < 0.01; *** – p < 0.001, ns – not significant.

Pattern eval. parameter	Factor	Verum		SNC		Treatment	Verum		SNC	
		F	*p*	F	*p*		n	Mean	n	Mean
Count	Patient	18.47	<0.0001 ***	20.72	<0.0001 ***	Individual	117	8.74 a	117	10.45 a
	Treatment	1.34	0.2609 ns	3.07	0.0278 *	*Pulsatilla*	117	9.53 a	117	9.23 b
	Interaction	1.60	0.0178 *	1.00	0.4661 ns	*Nat-mur.*	117	9.21 a	117	9.48 ab
						Placebo	117	9.51 a	117	8.99 b
Average size	Patient	2.22	0.0103 *	4.33	<0.0001 ***	Individual	117	24.60 x 10^3 a	117	24.53 x 10^3 a
	Treatment	0.85	0.4658 ns	0.67	0.5678 ns	*Pulsatilla*	117	22.32 x 10^3 a	117	24.76 x 10^3 a
	Interaction	1.22	0.1867 ns	1.06	0.3838 ns	*Nat-mur.*	117	25.04 x 10^3 a	117	24.35 x 10^3 a
						Placebo	117	24.85 x 10^3 a	117	26.84 x 10^3 a
Perimeter	Patient	6.86	<0.0001 ***	6.34	<0.0001 ***	Individual	117	16.90 x 10^2 a	117	16.59 x 10^2 a
	Treatment	0.91	0.4363 ns	0.79	0.4994 ns	*Pulsatilla*	117	15.27 x 10^2 a	117	16.56 x 10^2 a
	Interaction	1.35	0.0914 ns	1.03	0.4173 ns	*Nat-mur.*	117	17.39 x 10^2 a	117	16.05 x 10^2 a
						Placebo	117	17.45 x 10^2 a	117	18.25 x 10^2 a
Major	Patient	6.64	<0.0001 ***	5.69	<0.0001 ***	Individual	117	169.49 a	117	169.16 a
	Treatment	1.32	0.2674 ns	0.67	0.5690 ns	*Pulsatilla*	117	154.59 a	117	173.50 a
	Interaction	1.28	0.1339 ns	1.18	0.2280 ns	*Nat-mur.*	117	177.25 a	117	171.87 a
						Placebo	117	180.08 a	117	187.44 a
Minor	Patient	1.68	0.0675 ns	3.81	<0.0001 ***	Individual	117	63.66 a	117	62.48 a
	Treatment	0.81	0.4912 ns	0.70	0.5526 ns	*Pulsatilla*	117	59.00 a	117	67.20 a
	Interaction	1.58	0.0203 *	1.24	0.1658 ns	*Nat-mur.*	117	63.13 a	117	63.60 a
						Placebo	117	62.49 a	117	66.50 a
Circularity	Patient	3.86	<0.0001 ***	4.23	<0.0001 ***	Individual	117	0.57 a	117	0.57 a
	Treatment	1.38	0.2497 ns	1.15	0.3284 ns	*Pulsatilla*	117	0.59 a	117	0.55 a
	Interaction	1.17	0.2330 ns	1.23	0.1708 ns	*Nat-mur.*	117	0.57 a	117	0.57 a
						Placebo	117	0.56 a	117	0.54 a
Solidity	Patient	10.57	<0.0001 ***	6.60	<0.0001 ***	Individual	117	0.69 ab	117	0.70 a
	Treatment	1.42	0.2351 ns	0.78	0.5028 ns	*Pulsatilla*	117	0.70 a	117	0.68 a
	Interaction	1.40	0.0686 ns	1.47	0.0416 *	*Nat-mur.*	117	0.69 ab	117	0.70 a
						Placebo	117	0.68 b	117	0.68 a
Feret	Patient	7.66	<0.0001 ***	6.34	<0.0001 ***	Individual	117	260.84 a	117	256.10 a
	Treatment	1.20	0.3079 ns	0.69	0.5581 ns	*Pulsatilla*	117	238.03 a	117	264.24 a
	Interaction	1.43	0.0560 ns	1.09	0.3380 ns	*Nat-mur.*	117	270.20 a	117	256.42 a
						Placebo	117	276.41 a	117	282.71 a
Feret Angle	Patient	2.19	0.0115 *	1.91	0.0314 *	Individual	117	97.11 a	117	96.46 a
	Treatment	2.70	0.0453 *	2.43	0.0648 ns	*Pulsatilla*	117	96.20 a	117	95.57 a
	Interaction	1.33	0.0991 ns	0.87	0.6862 ns	*Nat-mur.*	117	90.61 b	117	90.33 b
						Placebo	117	92.24 ab	117	95.21 ab
Min Feret	Patient	2.55	0.0030 **	5.33	<0.0001 ***	Individual	117	119.75 a	117	117.62 a
	Treatment	0.82	0.4810 ns	0.58	0.6299 ns	*Pulsatilla*	117	110.53 a	117	122.49 a
	Interaction	1.78	0.0046 **	1.22	0.1863 ns	*Nat-mur.*	117	118.73 a	117	115.49 a
						Placebo	117	119.95 a	117	124.09 a

Table S5: *P*-values of one-way analyses of variance of individual blood samples, with the *treatment* factor of the particle analysis run on binary ROIs containing the target form of the green colour channel performed for the individual blood samples. Significances are marked in bold ($p < 0.05$). Blood samples responding to the treatments are marked with letter 'R'.
Legend: * – p < 0.05; ** – p < 0.01; *** – p < 0.001.

Pattern evaluation parameter	1	R2	3	4	5	R6	7	R8	R9	10	R11	R12	13
Count	0.3977	0.1491	0.1489	0.6738	0.8301	**0.0151***	0.2207	0.1181	0.6215	0.1648	**0.0221***	0.2335	0.5887
Average Size	0.6396	**0.0230***	0.1903	0.8144	0.6054	**0.0229***	0.3784	0.3553	0.6215	0.1310	0.1787	0.3201	0.5242
Perimeter	0.6430	**0.0227***	0.2295	0.8318	0.6263	**0.0181***	0.3789	0.3667	0.6412	0.2001	0.1940	0.2483	0.4238
Major	0.6237	**0.0226***	0.2904	0.8575	0.7067	0.0811	0.4115	0.1949	0.4250	0.4951	0.3108	0.0961	0.9241
Minor	0.6966	**0.0100***	0.0566	0.5527	0.7951	**0.0045****	0.3723	**0.0424***	0.4358	0.4697	0.1207	0.4620	0.7450
Circularity	0.3979	**0.0035****	0.0782	0.7458	0.7456	0.2352	0.2031	0.1436	**0.0205***	0.5103	0.2119	**0.0307***	0.8259
Solidity	0.7124	**0.0122***	0.0677	0.9132	0.6671	0.0949	0.1895	**0.0490***	**0.0147***	0.8004	0.4493	0.1309	0.9834
Feret	0.6475	**0.0181***	0.2634	0.8776	0.6616	**0.0230***	0.3330	0.2160	0.3522	0.3116	0.1889	0.1336	0.8278
FeretAngle	0.5522	0.2107	0.0973	0.1877	0.2005	0.6294	0.5277	**0.0119***	0.6827	0.0589	0.1538	0.1179	0.1808
MinFeret	0.7608	**0.0066****	0.0519	0.6275	0.7394	**0.0006*****	0.3219	**0.0378***	0.4082	0.3423	0.0951	0.4328	0.4996

Table S6: Correlations (Pearson's r values) between the blood count and clinical chemistry results and the pattern evaluation parameters for green channel images of region of interest containing the target forms (ROI). Strong correlations (r ≥ 0.70 or r ≤ -0.70) are indicated in bold.

	Count	Avr. size	Perimeter	Major	Minor	Circularity	Solidity	Feret	Feret Angle	MinFeret
Haemoglobin	**0.91**	**-0.84**	**-0.83**	**-0.84**	-0.68	0.60	0.68	**-0.83**	0.65	**-0.76**
Haematocrit	**0.92**	**-0.88**	**-0.88**	**-0.88**	**-0.77**	0.69	**0.77**	**-0.88**	**0.70**	**-0.84**
MCV	-0.59	0.67	**0.72**	**0.71**	0.55	-0.59	-0.67	**0.73**	**-0.72**	0.68
MCH	-0.50	0.65	**0.70**	0.69	0.58	-0.65	**-0.73**	**0.71**	**-0.74**	0.70
MCHC	0.20	0.07	0.08	0.06	0.24	-0.32	-0.32	0.08	-0.18	0.22
MPV	-0.42	0.24	0.23	0.25	0.37	-0.34	-0.23	0.24	-0.22	0.32
Erythrocytes	**0.86**	**-0.84**	**-0.85**	**-0.85**	**-0.71**	0.67	**0.76**	**-0.86**	**0.73**	**-0.81**
Leukocytes	0.06	-0.24	-0.28	-0.20	0.16	0.10	0.30	-0.23	0.40	-0.06
Neutrophils %	0.14	-0.34	-0.31	-0.30	-0.18	0.22	0.29	-0.29	0.33	-0.24
Neutrophils #	0.08	-0.31	-0.32	-0.25	0.09	0.14	0.33	-0.28	0.42	-0.11
Lymphocytes %	-0.04	0.19	0.16	0.14	0.09	-0.08	-0.08	0.15	-0.13	0.13
Lymphocytes #	0.07	-0.11	-0.16	-0.12	0.11	0.10	0.23	-0.14	0.27	-0.02
Monocytes %	-0.35	0.50	0.47	0.45	0.19	-0.20	-0.43	0.45	-0.49	0.29
Monocytes #	-0.30	0.25	0.20	0.24	0.28	-0.08	-0.13	0.22	-0.08	0.19
Eosinophils %	0.01	-0.01	-0.01	0.03	0.12	-0.25	-0.17	0.00	-0.06	0.05
Eosinophils #	-0.05	-0.05	-0.06	0.00	0.19	-0.24	-0.09	-0.03	0.04	0.06
Basophils %	0.12	0.04	0.08	0.06	-0.03	-0.07	-0.21	0.07	-0.28	0.00
Basophils #	0.16	-0.08	-0.05	-0.06	-0.05	0.03	-0.06	-0.06	-0.13	-0.08

RDW %	**-0.72**	0.69	**0.71**	**0.71**	0.48	-0.41	-0.53	**0.71**	-0.60	0.60
IG %	-0.40	0.18	0.23	0.24	0.02	-0.06	-0.10	0.25	-0.27	0.14
IG #	-0.41	0.16	0.21	0.22	0.05	-0.08	-0.09	0.24	-0.24	0.14
Normoblasts %	-0.35	0.33	0.33	0.31	0.10	0.04	-0.09	0.33	-0.25	0.20
Normoblasts #	-0.36	0.33	0.33	0.31	0.10	0.04	-0.09	0.33	-0.25	0.20
Platelets	0.25	-0.19	-0.19	-0.19	-0.41	0.35	0.26	-0.19	0.06	-0.35
Sodium	-0.04	-0.14	-0.09	-0.08	-0.12	0.09	0.15	-0.08	-0.02	-0.12
Potassium	-0.15	0.43	0.44	0.39	0.22	-0.20	-0.34	0.42	-0.38	0.37
Calcium	0.02	0.38	0.37	0.35	0.15	-0.11	-0.27	0.35	-0.35	0.27
Creatinine	0.37	-0.14	-0.11	-0.14	-0.16	0.07	0.03	-0.12	0.02	-0.12
Urea	0.32	0.04	0.04	0.03	0.20	-0.17	-0.18	0.04	0.00	0.16
Uric acid	-0.12	0.34	0.35	0.36	0.25	-0.22	-0.26	0.34	-0.40	0.29
Bilirubin, Total	-0.26	0.43	0.44	0.41	0.13	-0.35	-0.43	0.41	-0.40	0.31
AST	-0.67	**0.85**	**0.86**	**0.84**	0.43	-0.45	-0.64	**0.84**	**-0.78**	0.64
ALT	-0.24	0.29	0.32	0.33	-0.10	-0.11	-0.24	0.31	-0.29	0.10
GGT	-0.42	0.59	0.67	0.61	0.13	-0.32	-0.57	0.64	**-0.74**	0.43
Alkaline phosphatase	-0.44	0.21	0.26	0.29	0.58	-0.56	-0.43	0.29	-0.12	0.48
LDH	-0.68	**0.70**	0.68	**0.73**	**0.74**	**-0.74**	-0.67	**0.71**	-0.51	**0.73**
Current glucose	-0.09	0.05	0.03	0.10	0.30	-0.19	-0.08	0.07	0.11	0.17
Albumin	0.21	0.22	0.21	0.17	0.21	-0.18	-0.25	0.18	-0.19	0.25

Legend: MCV – mean corpuscular volume, MCH – mean corpuscular haemoglobin, MCHC – mean corpuscular haemoglobin concentration, MPV – mean platelet volume, RDW % – red cell distribution width, IG – immunoglobulins, AST – aspartate aminotransferase, GGT – γ-glutamyl transferase, LDH – lactate dehydrogenase, # – absolute values, % – percentage.
